# A Race Against Time: Rapidly Progressing Pulmonary Kaposi Sarcoma

**DOI:** 10.7759/cureus.40019

**Published:** 2023-06-05

**Authors:** Jasmine Tidwell, Sarah Van Antwerp, Zade A Bihag

**Affiliations:** 1 Internal Medicine, University of Connecticut Health, Hartford, USA

**Keywords:** highly active antiretroviral therapy, acquired immunodeficiency syndrome, human immunodeficiency virus, human herpes virus type 8, pulmonary kaposi sarcoma, kaposi sarcoma

## Abstract

Kaposi sarcoma (KS) is an acquired immunodeficiency syndrome-defining condition that primarily manifests as mucocutaneous lesions; however, other organs have been implicated in disseminated disease. Fortunately, since the development of antiretroviral therapy, the incidence of KS among patients with human immunodeficiency virus has significantly declined. We report an atypical case of a rapidly progressing pulmonary KS to highlight the importance of prompt recognition of this condition, which can be challenging to distinguish from other pulmonary infectious diseases in immunocompromised individuals, as well as discuss the current treatment for this disease.

## Introduction

Kaposi sarcoma (KS) is a malignancy caused by human herpesvirus 8 (HHV8), which was first identified in its association with human immunodeficiency virus (HIV) and acquired immunodeficiency syndrome (AIDS) in the 1980s [1]. The virus, also known as Kaposi sarcoma herpesvirus (KSHV), is a double-stranded DNA gammaherpesvirus that typically targets vascular and endothelial cells, though the pathogenesis is poorly understood [1-4]. KS is recognized as an opportunistic malignancy most commonly observed in patients with CD4 counts less than 200 [5,6]. The most well-known manifestation of the malignancy is the pigmented subcutaneous skin nodules [1-3]. While 80-90% of KS cases involve mucocutaneous lesions, KS has also been observed in the lungs, gastrointestinal tract, liver, bone, and lymph nodes [5-7]. However, since the development of antiretroviral therapy (ART), there has been an overall decline in the incidence of KS among individuals with HIV [8]. Presenting symptoms can include fatigue, weight loss, night sweats, and generalized adenopathy [1].

## Case presentation

**Figure 1:**
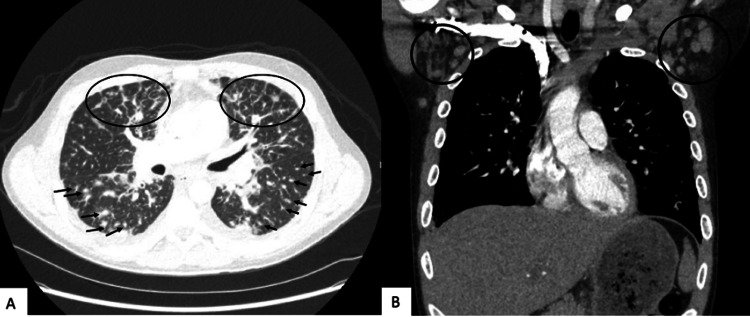
Computerized tomography of the chest with extensive bilateral lung perilymphatic nodularity (A, arrows), nodular interlobular septal thickening in a flame-shaped morphology (A, circles), and bilateral axillary lymphadenopathy (B).

We present the case of a 33-year-old male with a recent diagnosis of HIV/AIDS (CD4 383 mm^3^, viral load 22,100 copies/mL) who was non-compliant with bictegravir/emtricitabine/tenofovir (Biktarvy). He initially presented to the hospital due to shortness of breath, productive cough, and intermittent fever for several weeks. He also noted the development of raised, purple lesions on the left forearm and abdomen four months prior. A computerized tomography (CT) of the chest showed extensive bilateral lung perilymphatic nodularity, nodular interlobular septal thickening in a flame-shaped morphology (Figure 1A), and bilateral axillary lymphadenopathy (Figure 1B), suspicious for KS.

He was subsequently discharged from the emergency department due to hemodynamic stability with plans for outpatient follow-up with pulmonology. He was instructed to be compliant with his ART and prophylactic trimethoprim-sulfamethoxazole and valacyclovir.

**Figure 2:**
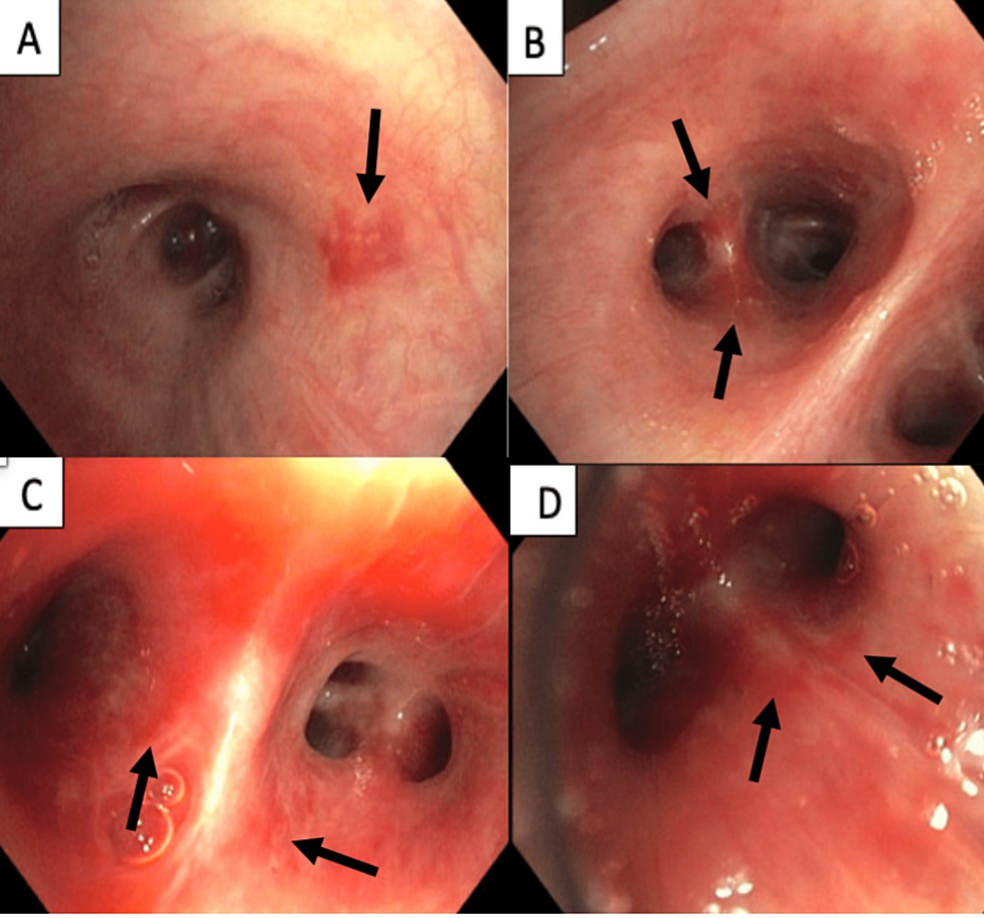
Flexible bronchoscopy revealing flat, macular, erythematous lesions from the mainstem bronchi (A) and subsegmental airways with more erythematous lesions bilaterally (B-D).

One month later, he underwent outpatient flexible bronchoscopy with biopsy, which revealed numerous macular, erythematous endobronchial lesions within the mainstem (Figure 2A) and bilateral subsegmental bronchi (Figure 2B-2D). Biopsy was positive for CD31, ERG, and HHV8, consistent with KS.

**Figure 3:**
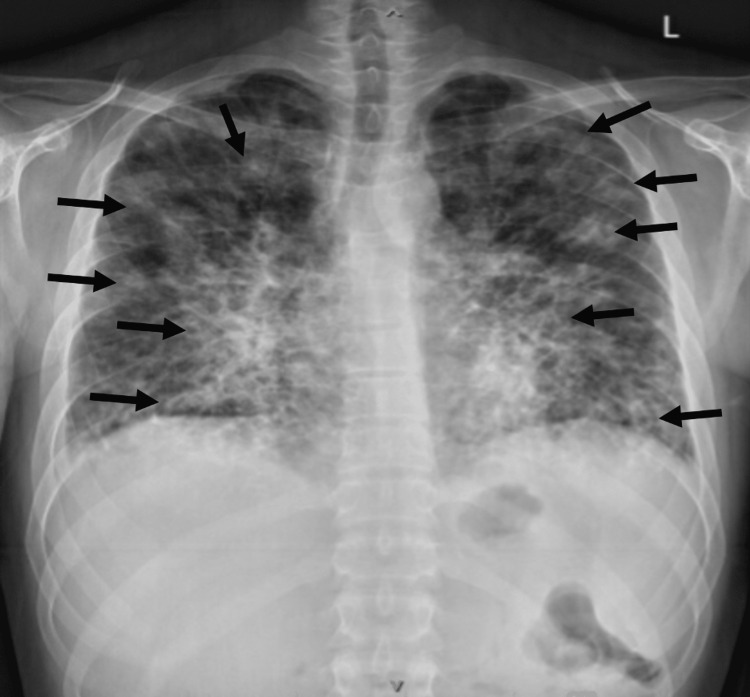
Chest X-ray with multifocal bilateral airspace opacities.

Three weeks later, he presented to the hospital due to worsening respiratory status and high-grade fevers. Upon arrival, respiratory rate was 30-40 breaths/minute, and heart rate was 100 beats/minute. Labs were significant for CD4 of 73 mm^3^, hemoglobin of 8.5 g/dL, platelet count of 74 × 10^3^/mL, elevated lactate dehydrogenase of 336 U/L, lactate of 2.2 mmol/L, and procalcitonin of 3.02 ng/mL. His cryptococcal antigen and respiratory panel were negative. Initial chest X-ray revealed multifocal bilateral airspace opacities (Figure 3).

**Figure 4:**
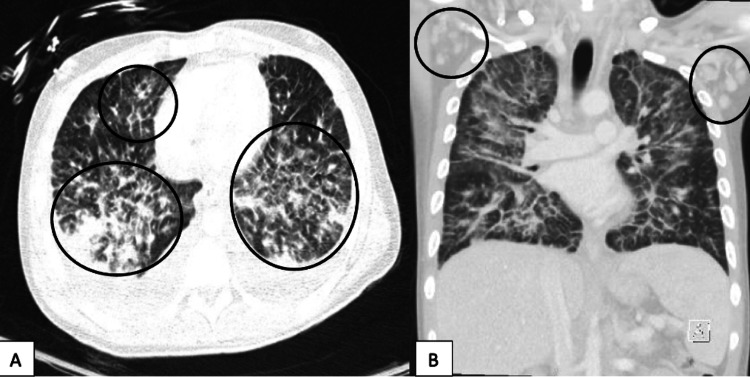
Computerized tomography of the chest with severe diffuse ground-glass opacities (A) and bilateral axillary lymphadenopathy (B).

CT of the chest revealed severe diffuse ground-glass opacities (Figure 4A) and bilateral axillary lymphadenopathy (Figure 4B).

Home Biktarvy along with prophylactic trimethoprim-sulfamethoxazole and valacyclovir were continued. Hematology/Oncology was consulted to treat KS, who advised ruling out an infectious component before starting chemotherapy.

His hospital course was quickly complicated by increasing oxygen requirements, for which he was intubated and underwent a repeat bronchoscopy to obtain a bronchoalveolar lavage for ruling out an underlying infection. Respiratory culture, cytology, acid-fast bacillus, *Pneumocystis jirovecii, Legionella pneumophila*, and fungal cultures were negative. He was extubated to a high-flow nasal cannula but continued with significant respiratory distress. Repeat CD4 was 70 mm^3^. As an infectious process was ruled out, Hematology/Oncology recommended initiating chemotherapy. However, after discussing his poor prognosis, he ultimately decided not to proceed with chemotherapy and was discharged to home hospice.

## Discussion

KS is an opportunistic malignancy, occurring in patients with a CD4 count less than 200 mm^3^ [3]. The incidence of the disease has declined substantially since it was first identified in the 1980s due to the advancement and availability of ART and its prevention of HIV progression to AIDS [1,6,8]. In our case, the patient had been noncompliant with Biktarvy since his diagnosis two years prior and upon admission had a CD4 count of 73 mm^3^. The patient presented twice with vague pulmonary complaints initially attributed to a more likely cause, such as an infection. This prompted a complete infectious workup which led to a delay in diagnosing pulmonary Kaposi and, eventually, a delay in therapy.

The diagnosis of pulmonary KS can usually be confirmed through a combination of clinical, laboratory, radiography, bronchoscopy, and transbronchial biopsy [9,10]. Similar to our patient, the typical presentation usually includes fever, cough, and dyspnea [10]. The most common findings in chest radiography include bronchial wall thickening, nodules, pleural effusions, and Kerley B lines. Frequent findings on chest CT include interstitial thickening, parenchymal nodules, ground-glass opacities, mediastinal adenopathy, and pleural effusions [10]. A high-resolution CT scan is considered more helpful in leading to the diagnosis of the disease with higher sensitivity and specificity [10]. A presumptive diagnosis of pulmonary KS can be made if characteristic macular red or violet tracheobronchial KS lesions are identified with bronchoscopy [9]. Differentials include immune reconstitution inflammatory syndrome-associated KS; however, this is characterized by a reduction of at least 1 log10 of HIV-1 RNA and/or an increase of ≥50 cells/mm^3^ or ≥two-fold rise in baseline CD4+ cell count [11]. Our patient’s repeat CD4 decreased by 3 mm^3^.

There are two main treatment pillars for managing KS, namely, ongoing ART and concurrent chemotherapy agents such as liposomal doxorubicin and paclitaxel [3]. Some studies reported 85% survival in patients with non-pulmonary KS using this treatment regimen [4]. In patients with pulmonary involvement, airway protection presents an additional challenge for therapy [7,12,13]. While highly active ART and systemic chemotherapy are utilized, patients may also benefit from treatment modalities such as endoscopic laser resection, radiation therapy, or stent placement for airway maintenance [7,13].

While ART has played a role in preventing and treating KS, there is still much to be learned regarding further disease management. The pathogenesis of KS is still not well understood. One pathway which is being studied is vascular endothelial growth factor inhibition and the use of checkpoint inhibitors [1,3]. Prognosis is generally guarded. In one study, median survival in patients with pulmonary KS was 1.6 years; however, our patient’s course was six months [7]. Patients with advanced pulmonary KS, such as the patient described in this case report, would benefit from quick diagnosis and prompt initiation of chemotherapy to improve survival rates.

## Conclusions

Here, we present a unique case of rapidly progressing pulmonary KS. Pulmonary Kaposi has a median survival of 1.6 years; however, our case quickly progressed over the course of six months. Due to its increased morbidity, it is imperative to raise awareness for the potential pulmonary involvement of KS in patients with AIDS. As in our case, it is also important to rule out any underlying infectious process before starting chemotherapy. The early recognition of pulmonary KS is crucial to reach an early diagnosis and ensuring timely and successful treatment to prevent fatal outcomes.
